# Analysis of Grain Growth Behavior of Intermetallic Compounds on Plated Pure Sn for Micropump Solder Caps

**DOI:** 10.3390/ma18194602

**Published:** 2025-10-03

**Authors:** Hwa-Sun Park, Chang-Yun Na, Jong-Wook Kim, Woon-Seok Jung, Jae-Hyuk Park, Jong-Woo Lim, Youn-Goo Yang

**Affiliations:** 1 Department Electrical/Chemical Engineering, Sungkyunkwan University, Suwon 16419, Republic of Korea; nmrl-noinsung@naver.com (C.-Y.N.); jhpark@estek.co.kr (J.-H.P.); jwlim80@skku.edu (J.-W.L.); 2 Hojinplatech 91, Mongnae-ro 119, Danwon-gu, Ansan-si 15588, Republic of Korea; jukim@hojinplatech.com (J.-W.K.); wsjung@hojinplatech.com (W.-S.J.); 3 EST 103-14, Gajangsaneopseobuk-ro, Osan-si 18102, Republic of Korea

**Keywords:** solder cap, pure Sn, microbump, intermetallic compound, grain

## Abstract

We evaluated for the morphology and growth behavior of IMC grain according to number of reflows of solder cap pure Sn microbumps. In the structure of Ni barrier/Cu layer between Cu pillar and pure Sn, solder cap pure Sn on the top layer was analyzed for the behavior change of IMC grain according to the number of reflows. The height and diameter of the bumps on the wafer were designed to be 40 μm and 30 μm, respectively. The vertical structure of the microbump consisted of Ti/Cu (1000 Å/2000 Å), Cu pillar (20 µm), Ni barrier (3 µm), and Cu (1 µm). The overall height of the bump is about 40 μm. Additionally, the height of the solder cap pure Sn as the last layer is 20 μm. The diameter of the bump is 30 μm. It was formed using plating. After plating to solder cap Sn, it was finally formed for the microbump using reflow. Samples were prepared according to the number of reflows (1, 3, 5, 7, and 9). To observe the grain morphology of the IMC, the pure Sn on the upper layer (solder cap) was removed using SupraBond RO-22 etchant. In the removed state, the morphology of the IMC grain was evaluated to the inside surface of bump using SEM and a 3D scope. The average number of IMC grains decreased linearly during reflow cycles 1 to 5 and then gradually decreased during reflow cycles 7 to 10. The average surface area of IMC grains was 18.243 μm when reflow was performed once. The average surface area of IMC grains increased proportionally for reflow cycles 1 to 10. Based on the experimental results, when the count of reflow was performed more than 10 times, it was confirmed that the solder cap pure Sn was reduced by more than 50% due to the increase in the area of IMC grain.

## 1. Introduction

Recently, the increasing demand for high-specification semiconductors used in high-performance computing, artificial intelligence, 6G communications, and data communications has led to a demand for high integration, high density, and miniaturization of semiconductor packages [1,2,3,4]. To meet the demands of these semiconductors, the need for differentiated packaging technologies is growing. In particular, wire bonding and solder bumping are used to electrically interconnect mainboards and modules, PCBs and ICs, and wafers with other wafers. Solder bumping technology has long been the subject of extensive research to achieve high integration, high density, and miniaturization. It is applied to high-spec semiconductor packages to connect high-density electrodes of semiconductor devices or sensors. To improve semiconductor packaging, solder bumping bonding methods are being researched, including various materials and processes [5,6].

Microbumps, which dramatically reduce bump size and spacing, are being used as a method of interconnecting electrodes to achieve high-density and high-miniaturization semiconductor packages. According to a recent announcement by Yole, package structures are classified based on the diameter of the solder bumps used in ball grid arrays (BGAs) and chip scale packages (CSPs). The BGA features solder bumps with diameters of 400–500 μm, which are relatively large, and CSP refers to solder bumps with diameters of 250–400 μm for use in packaging technology similar to the size of a chip. Microbumps refer to solder bumps with a diameter of 10–100 μm and are much smaller than CSPs and can be used in high-density packaging or 3D semiconductor packaging. For example, high bandwidth memory (HBM) is a high-performance RAM interface for 3D stacked DRAM using advanced packaging technology. The microbumps used in HBM have an average size of 20 μm and are used for electrical connections between chips and between chips and substrate [7,8,9,10].

The primary formation technology of microbumps is plating with indium, tin, or tin alloy materials on specific areas of a substrate. Electroplating technology for fabricating SnAg or Sn solder bumps allows easier and faster pattern production as the pattern size becomes smaller, and considerable research has been conducted. Packaging technology utilizing electroplating bumps has lower impedance than conventional wire bonding methods, can reduce package size to a single die, and offers excellent heat dissipation [11,12,13,14].

A recent method for forming microbumps is to plate Cu pillars on the top of larger bumps. Micro Cu pillars significantly improve the I/O density between chips by reducing the gap between bumps. However, for microsolder bumps formed on Cu pillars, the plating thickness is small, typically around 20 to 60 μm, increasing the intermetallic compound (IMC) volume in both the horizontal and vertical directions. This reduces the shear strength of the solder bump [15,16,17,18]. During soldering, IMCs are formed at the joint according to temperature and time and exhibit significant variation. In particular, excessive growth can negatively impact the joint, increasing brittleness and reducing electrical properties [19,20,21,22].

Because the amount of solder cap formed on the upper layer of the Cu pillar is limited, excessive IMC growth decreases layer strength. To resolve this, an additional barrier layer has been inserted between the Cu pillar and the solder cap to suppress the growth of IMCs by controlling the interface. A Ni layer applied as a barrier to suppress IMC growth is formed through ENEPIG surface treatment slowed IMC growth at the joint interface. Based on that, a Ni layer has been inserted between a Cu pillar and solder cap [23,24,25]. The addition of Cu@Sn@Ag (CSA) core-shell particles to SAC305 solder joints significantly enhances their overall performance by refining the microstructure, increasing corrosion resistance, and improving mechanical properties [26]. The micro-roughness of microbumps can be precisely analyzed on many samples using a 3D microscope. [27]

So far, the reviewed papers are mainly studying IMC according to temperature for SnAgCu and SnAg. Recently, in HBM and 2.5D/3D packages, it is being applied to an electrically interconnection method using solder cap Sn through plating. It implements a fine pitch and is widely used in high-spec packages. In particular, in the case of microbumps, since the area and size of the bumps are small, a close analysis of the IMC according to the number of reflows is required. Solder joints in high-spec packages are processed with a large number of reflows. At this time, it is important to closely observe for IMC the inside of the microbump.

Previous bump studies were focused mainly on observing the external changes of IMC in only a single section. As shown in the papers in [17,23], the electrical and physical verification for solder joints of microbumps was analyzed by cross-sectional views. Against this background, we will introduce a method to observe the behavior of IMC inside the bump.

Based on the structure of the Ni barrier formed between the Cu pillar and pure Sn, we observed how the behavior of IMC grain changes according to reflow. In order to find out the behavior of IMC grains inside the bump, pure Sn, a solder cap layer was removed using etching method. The shape, size, and area of IMC grains were assessed according to the number of reflows using a 3D microscope and an SEM.

## 2. Experiment

### 2.1. Manufacturing of a Pure Sn Solder Cap Bump with a Height of 40 μm and a Diameter of 30 μm

A microbump made of a structure with a height of 40 μm and a diameter of 30 μm is manufactured. The fabricated samples showed various changes depending on the number of reflow cycles. We observed changes in the growth morphology of IMCs generated in Sn and Cu layer structures in the plane and the side surface depending on the number of reflow cycles. Basically, temperature-dependent IMCs grow in grains in horizontal and vertical directions. To three-dimensionally elucidate the IMC growth mechanism of pure Sn, we removed the Sn formed during wet etching. After the removal, the side surface and plane morphology were analyzed using an SEM (Philips ESEM XL30, Philips, Cambridge, MA, USA) and a 3D scope (Keyence VK-X3050, Keyence, Seoul, Republic of Korea) and the growth mechanism was quantified.

First, a microbump with a vertical structure of 40 μm in height and 30 μm in diameter on the wafer was designed by layer and manufactured. The vertical structure of the bump was formed on a Si wafer with Ti/Cu 1000 Å/2000 Å, Cu pillar 20 μm, Ni barrier 3 μm, and Cu 1 μm. Then, the top-most solder cap layer was formed by plating with pure Sn at a diameter of 30 μm and a height of 20 μm. This pure Sn was then removed using a solder cap-specific etchant. The fabricated samples were analyzed for IMC morphology, grain count, area, and size on the side surface and planes according to reflow conditions.

**Figure 1:**
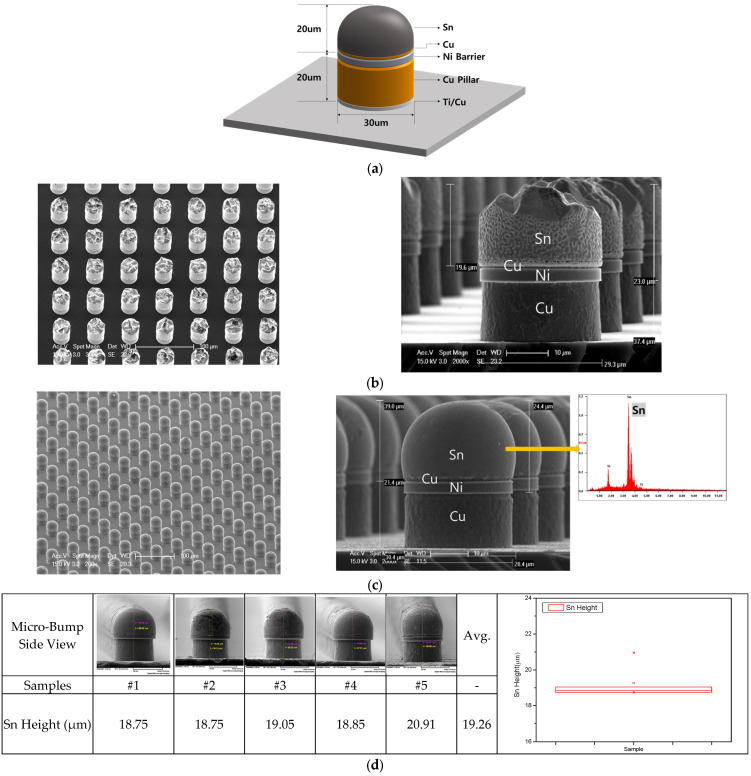
Design and fabrication of a pure Sn bump with a diameter of 30 μm. (**a**) A vertical structure of microbumps; (**b**) SEM images of the planar and the side surface after top layer pure Sn plating; and (**c**) the side surface image of a 30 μm diameter bump manufactured after reflowing (SEM side view). (**d**) Height of pure Sn after reflowing (SEM side view).

Figure 1a shows the vertical structure of a Sn bump with a height of 40 μm and a diameter of 30 μm. Figure 1b shows SEM shape of the planar and a side surface manufactured through plating before reflowing. The diameter and spacing of the bumps on wafer were 30 μm and 55 μm, respectively. The fabrication process involved depositing 1000 Å of Ti/2000 Å of Cu on a wafer, applying a 40 μm-thick dry film, and then opening a via using a photolithography process. A 20 μm Cu layer, a 3 μm Ni layer, and a 1 μm Cu layer were formed in the opened via hole. Finally, 20 μm-high pure Sn was formed by electroplating. After removing the dry film, the Ti and Cu were etched away. The electroplating process was performed at a distance of 155 mm from the electrodes and 65 mm from the cathode. Under current density 9ASD, temperature 30 °C, and 3.5 Lt/min, Sn was plated at a thickness of 20 μm [27]. Figure 1c is a vertical SEM image of a bump with a height of 40 μm and a diameter of 30 μm manufactured by the reflow process under the same conditions as for the sample of Figure 1b. The Sn bump formed by plating was performed after applying flux to the bump before reflowing. Reflow was performed 1, 3, 5, 7, and 10 times for each of the 5 samples prepared. Reflow was carried out in five stages as follows: 1st rising section (0–50 s @ 170 °C), preheating section (50–200 s @ 180 °C), 2nd rising section (200–250 s @ 240 °C), reflow section (250–300 s @ 246 °C), and cooling section (300–350 s @ 30 °C). The manufactured samples were prepared according to the number of reflow cycles. Figure 1d shows the height of pure Sn using a SEM side view after reflowing. The average Sn height of five samples is approximately 19.26 μm. The distribution is shown in the box plot on the right.

### 2.2. Wet Etching Experiment of a Solder Cap Sn Layer

For the top-layer bumps containing both IMC and pure Sn, we performed wet etching with Hojin Platech SupraBond RO-22 (Seoul, Republic of Korea) to remove the pure Sn. This strongly acidic organic acid solution strips tin from raw metals such as copper, copper alloys, nickel, and nickel alloys through a deposition process. It is suitable for both immersion and spray processes and does not generate the exothermic reaction that hinders the use of most common strippers. Furthermore, it selectively strips tin, leaving an intermetallic layer of copper and tin alloy that minimizes corrosion of the raw metals and stainless steel.

The reflow process conditions were as shown in Table 1. The Sn bump sample 1 was reflowed once, Sn bump sample 2 was reflowed three times, Sn bump sample 3 was reflowed five times, Sn bump sample 4 was reflowed seven times, and Sn bump sample 5 was reflowed ten times. The Sn etching method was as follows: first, 200 mL of SupraBond RO-22 etchant was added to the etching bow and stirred at 100 rpm for 5 min. The prepared bump samples were dipped in the etching bow to etch only the Sn at the top of the bump of the solder cap. The etched samples were sonicated in acetone for 5 min and in ethanol for 5 min and then rinsed in deionized water for 1 min.

### 2.3. Analysis

**Figure 2:**
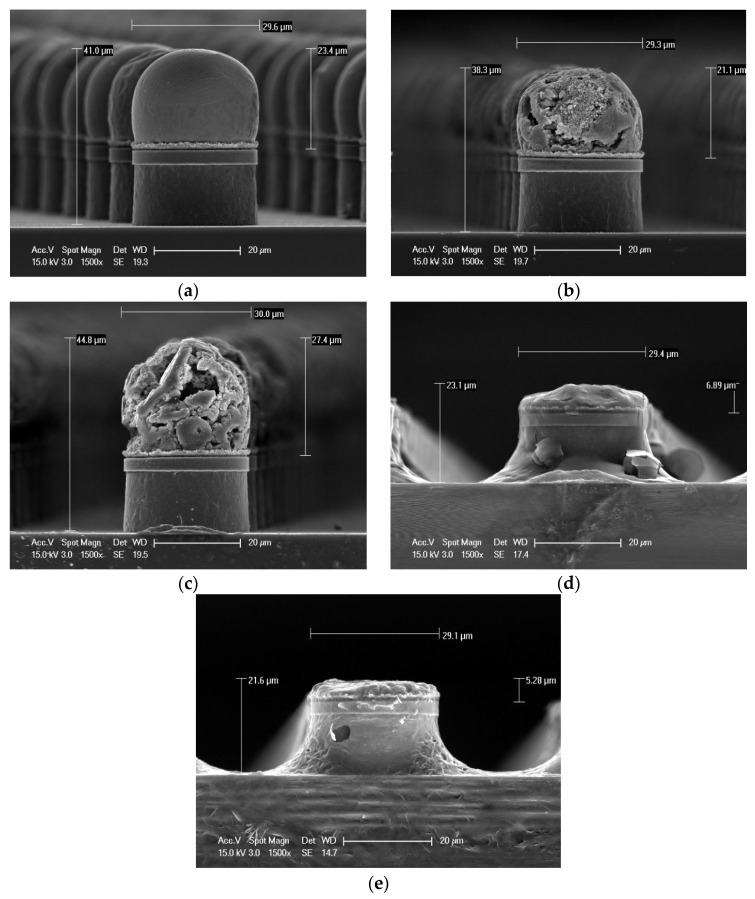
The side surface of the solder cap Sn layer after wet etching for different times: (**a**) 0 min, (**b**) 5 min, (**c**) 10 min, (**d**) 15 min, and (**e**) 20 min.

Figure 2 shows an experiment to determine the optimal etching time to remove only Sn using a sample of solder cap Sn bump 1. The etching times were 0, 5, 10, 15, and 20 min. After etching the solder cap Sn, the side surface at each time point was analyzed using SEM. Figure 2a shows the side surface shape before etching. At 5 min of etching, l removal of material can be seen at the outer surface of the solder cap Sn. At 10 min, removal proceeded toward the inside of the bump. At 15 min, almost all the cap Sn was etched, although some remained on the top surface of the bump. In the sample etched for 20 min, all Sn was removed. A 1 μm Cu layer formed on the Cu pillar/Ni barrier during reflow. The Sn-etched sample was immersed in acetone and ethanol and then sonicated for debris removal. It was confirmed that SupraBond RO-22 from Hojin Platech can selectively strip tin to leave an intermetallic layer.

**Figure 3:**
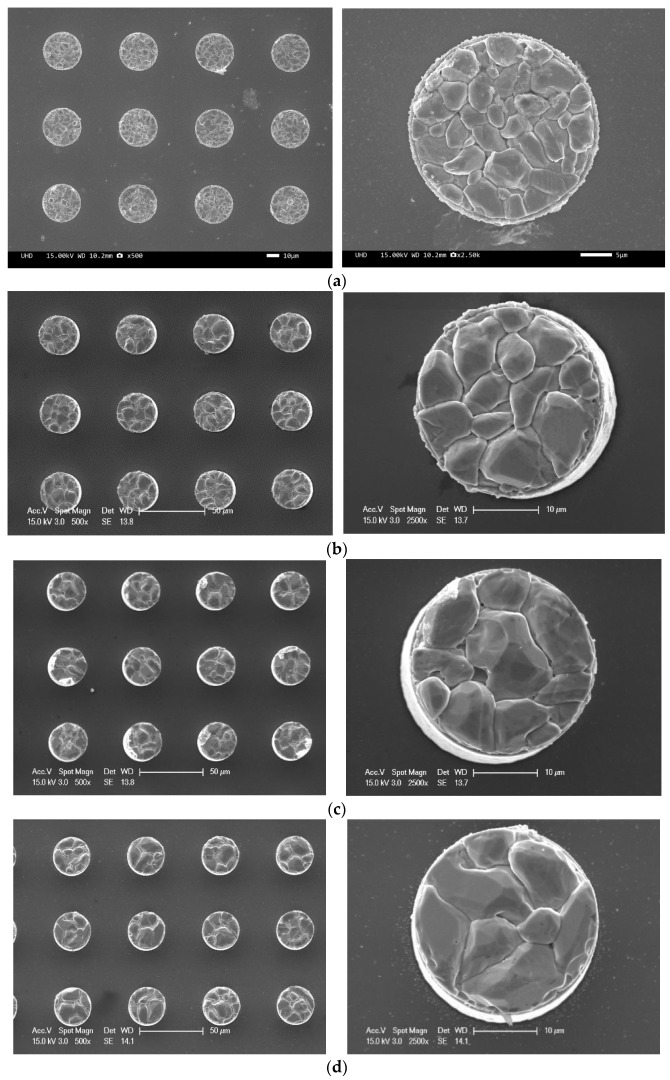

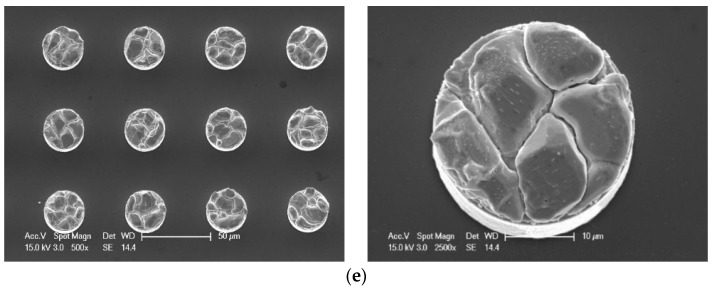
Planar SEM images of wet-etched bumps for five samples: (**a**) 1 reflow cycle, (**b**) 3 reflow cycles, (**c**) 5 reflow cycles, (**d**) 7 reflow cycles, and (**e**) 10 reflow cycles.

Figure 3 shows the planar SEM views of the etched solder cap Sn layer for the five samples presented in Table 1. For each sample, 12 bumps were imaged. The left image of Figure 3a shows SEM images of each of the 12 bumps, and the right image shows the surface condition of one of the bumps, enlarged. The grain and grain shape can be confirmed. Figure 3b–e also compare the surface condition according to each condition. The grain shapes of the IMC, an alloy of Sn and Cu, were irregular and small. With one reflow cycle, the plane view showed small, densely packed bumps. With three reflow cycles, slightly larger grain sizes were observed due to combinations of small IMC grains. Five reflow cycles showed a smaller number of even larger IMC grains. This pattern continued with additional reflow cycles. In brief, as the number of reflow cycles increased, the distance and depth of the curvature between the IMC grains increased.

**Figure 4:**
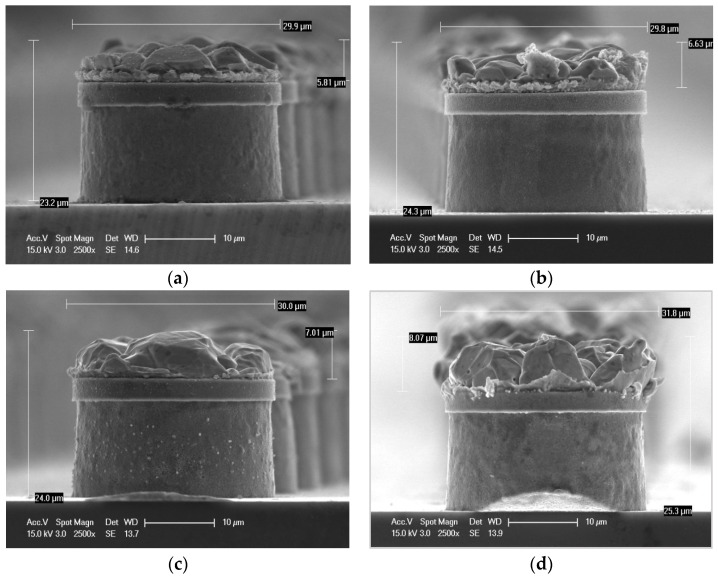
SEM images of the side view of the bumps with only Sn removed. (**a**) After 3 reflow cycle (**b**) After 5 reflow cycle (**c**) After 7 reflow cycle (**d**) After 10 reflow cycle.

Figure 4 compares the side surface SEM morphology of each sample with the planar shape according to the conditions in Figure 3. The side surface SEM results show that the Cu and Sn alloy layers formed on the Ni barrier layer were etched for each sample. Even when observed in the side surface, the size and number of IMC grains clearly change. The overall size of the IMC grain shape gradually increases from the 3rd to 10th reflow cycle, and the grain shape varies irregularly. A characteristic feature from the side surface SEM analysis is that the size and thickness of the IMC grains clearly increase. When reflowed 3 times, the thickness of the IMC layer is approximately 5 μm, but when reflowed 10 times, the thickness increases to more than 8 μm, the grain shape has a clear distinction between the valleys, and the height between the valleys increases. Additionally, the surface area of each grain increases as the number of reflows increases.

**Figure 5:**
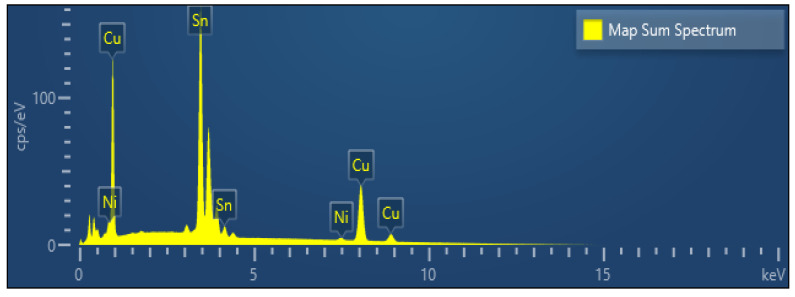
Atomic percentages of microbump mapping data.

Figure 5 shows a compositional analysis of the surface condition of the Sn-etched microbump. The results of the compositional analysis are shown in Table 2. As shown in Table 2, atomic composition analysis was performed using EDS on the plane of the Sn-etched microbump. If Sn had been etched to a smaller degree, the Sn component would have been dominant. However, as shown in the table, the detection of Cu, Sn, and Ni indicates that all solder cap Sn was etched. In addition, the detection of mainly Cu (40.56 wt%) and Sn (58.34 wt%) components represent an oxide alloy.

**Figure 6:**
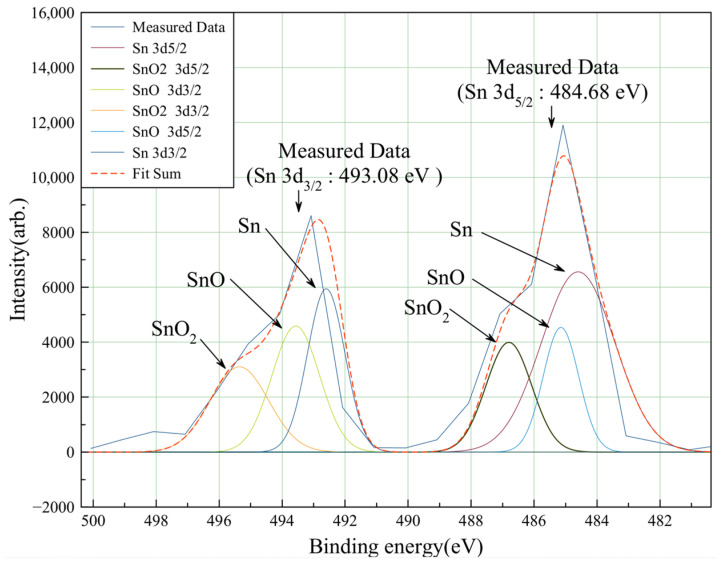
Analysis of Sn XPS scan data on microbumps with an etched Sn layer. Fitting the oxide composition data of Sn 3d_5/2_ and Sn 3d_3/2_ using measured Sn 3d scan data.

Figure 6 analyzes the oxide composition of the Cu and Sn components in Figure 5. Figure 6 is the X-ray photoelectron spectroscopy spectrum (model K-alpha plus) of Sn 3d on the top surface of the wet-etched bump. Fit of the oxide composition data of Sn 3d_5/2_ and Sn 3d_3/2_ using measured Sn 3d scan data. As shown in Figure 6, the measured Sn 3d scan data are used to fit the oxide composition data of Sn 3d_5/2_ and Sn 3d_3/2_. The binding energy of 484.68 eV corresponds to Sn 3d_5/2_, and the binding energy of 493.08 eV corresponds to Sn 3d_3/2_. The XPS mapping results for the measured results of Sn 3d_5/2_ and 3d _3/2_ confirmed oxides in the forms of SnO and SnO_2_.

## 3. Results and Discussion

**Figure 7:**
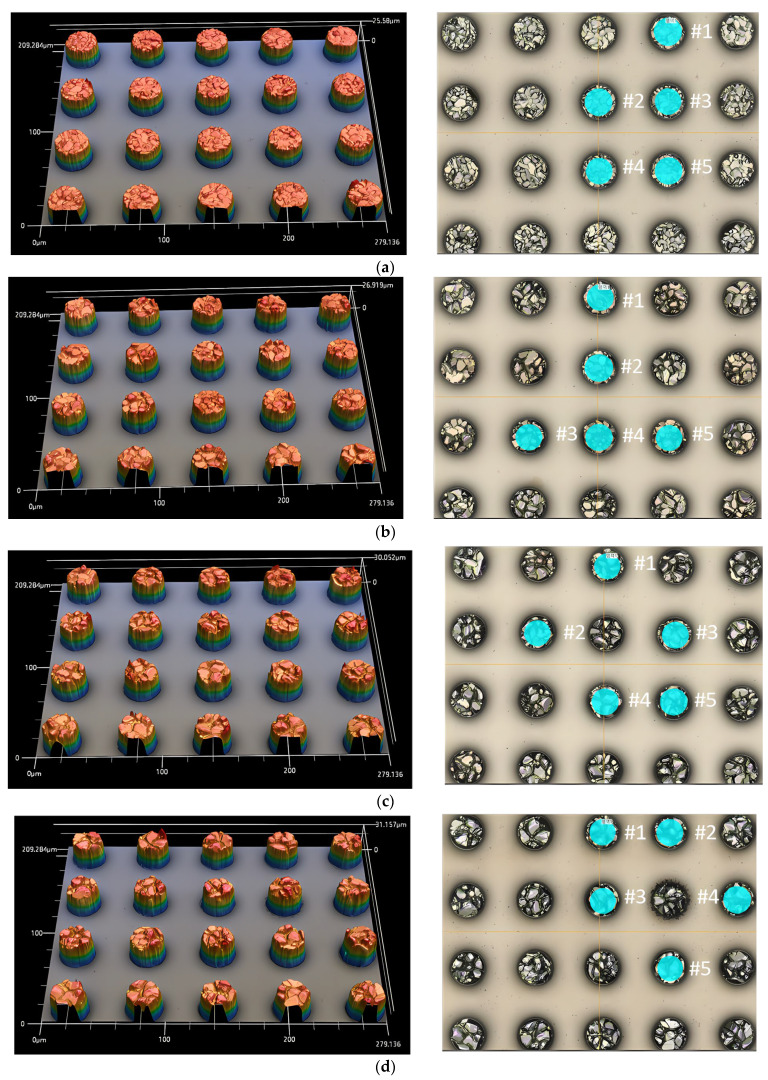

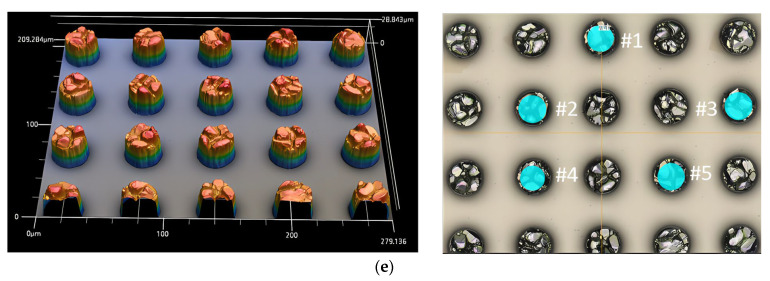
Planar morphology of Sn-etched IMC using 3D microscopy: (**a**) one reflow cycle, (**b**) three reflow cycles, (**c**) five reflow cycles, (**d**) seven reflow cycles, and (**e**) 10 reflow cycles.

Figure 7 shows the grain surface morphology and intergrain waviness depth of IMC according to sample conditions using a Keyence VK-X3050 3D microscope (Keyence VK-X3050, Keyence, Seoul, Republic of Korea). It was confirmed that the shape and number of grains varied depending on the number of reflow cycles.

Figure 7a is a 3D microscope photograph of the surface of the Sn-etched bumps after one reflow cycle. Five bumps were randomly selected from the twenty bumps for each condition. The five-bump planes were designated as circular areas (blue), and the roughness (Ra, Rz) for each area was measured. Similarly, the grain surface shape, number, and roughness were analyzed for the samples that were reflowed 3 times (Figure 7b), 5 times (Figure 7c), 7 times (Figure 7d), and 10 times (Figure 7e).

Table 3 shows the intergranular roughness of the IMC grain measured using a 3D microscope (Keyence VK-X3050, Keyence, Seoul, Republic of Korea). for the bumps sampled in Figure 7. Sa and Sz can be measured as surface roughness (Sa·Sz) within a designated area in a 3D plane using a Keyence VK-X3050 3D microscope. Sa represents the average surface roughness, and Sz represents the maximum height roughness of the surface.

Grains of IMC grow by combining with each other according to the number of reflow cycles, and curvature results between the newly combined grains. As shown in Table 3, the roughness was measured according to the conditions and is correlated with the shear strength of the bump [27]. In reality, the shear strength of the bump increases from the initial reflow cycle number, but the shear strength decreases with reflow cycling. Through this, the IMC growth thickness and curvature greatly affect the shear strength. The IMC growth characteristics are very different depending on the diameter and height of the bump, material, and temperature.

**Figure 8:**
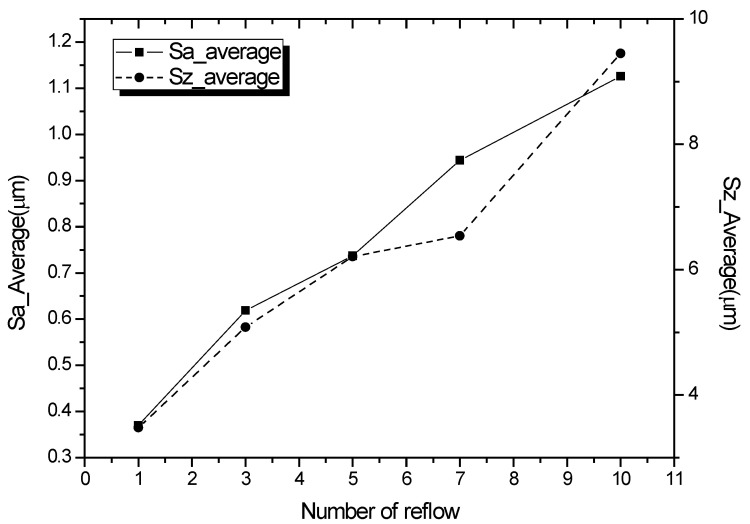
Average Sa and Sz according to reflow cycle.

Figure 8 compares the average Sa and Sz according to reflow for the measured data in Table 4. As in the Sa graph, the roughness increases proportionally as the number of reflow cycles increases. The average Sa is 0.370 μm at reflow 1 but increases linearly by almost three times to 1.126 at reflow 10. This can be confirmed by increase in depth between the valleys as the grain sizes of the IMC merge and increase in size.

**Figure 9:**
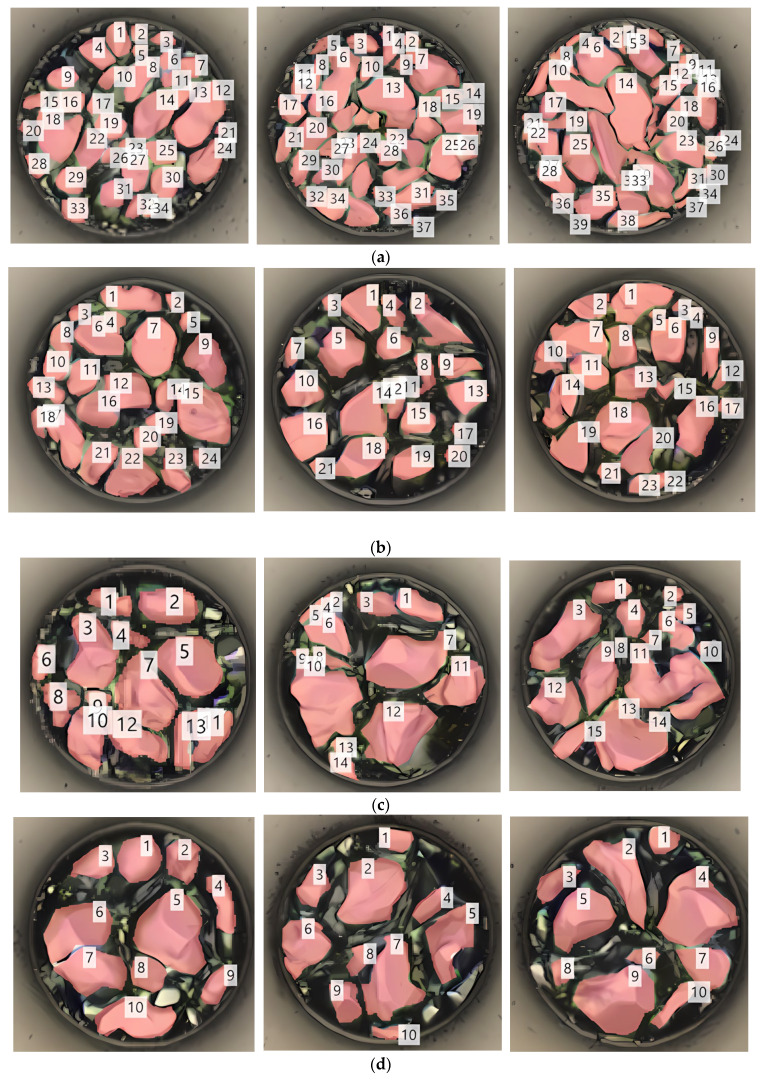

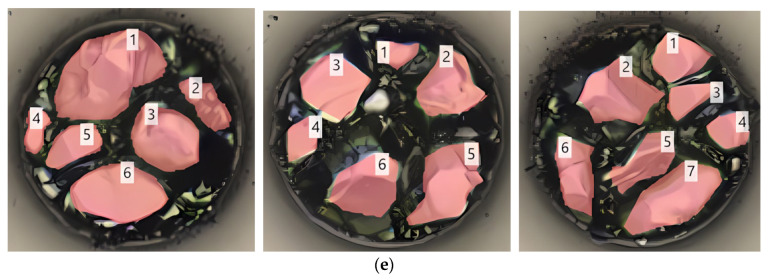
Surface area and number of IMC grains formed on a bump using a 3D microscope: (**a**) number of grains after 1 reflow, (**b**) number of grains after 3 reflows, (**c**) number of grains after 5 reflows, (**d**) number of grains after 7 reflows; and (**e**) number of grains after 10 reflows.

**Figure 10:**
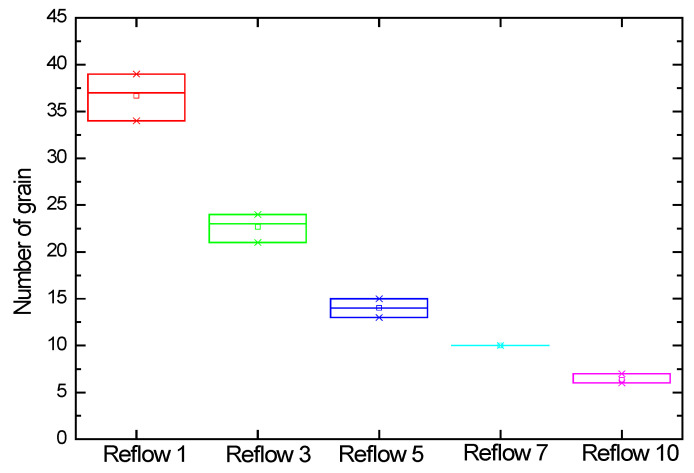
Number of IMC grains formed on the bump using a 3D microscope.

Figure 9 analyzes the number of grains for each sample captured by the 3D scope. Table 4 counts the grains formed on the bump plane according to the reflow conditions. For three bumps, the average number of grains for 1 reflow was 36.67. For 3 reflows, the average number of grains was 22.67. For 5 reflows, the average number of grains was 14. For 7 reflows, the average number of grains was 10. For 10 reflows, the average number of grains was 6.33. As shown in Figure 10, the number of grains decreased rapidly and proportionally from 1 to 5 reflows but gradually decreased from 7 to 10 reflows.

**Figure 11:**
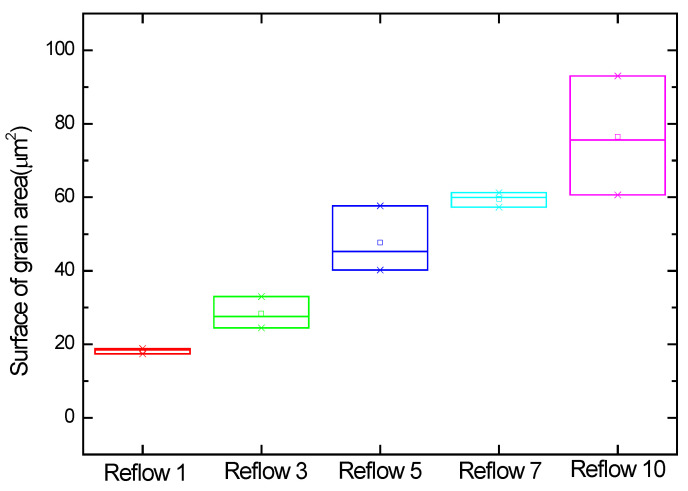
Surface area of IMC grains formed on top of the bump using 3D microscopy.

Table 5 shows the surface area analysis of grains formed on the bump plane. Keyence VK-X3050 is a laser microscope that uses triple scanning to analyze the surface area by measuring the three-dimensional shape and surface of a target object in a non-contact manner [27]. The average surface area of the 1 reflow was measured as 18.243 μm^2^, the average surface area of 3 reflows was 23.325 μm^2^, and the average surface area of 5 reflows was 47.669 μm^2^. The average surface area of 7 reflows was measured as 59.506 μm^2^, and the average surface area of 10 reflows was measured as 76.431 μm^2^. As shown in Figure 11, the average surface area of the IMC grains formed under each condition increased proportionally overall.

**Figure 12:**
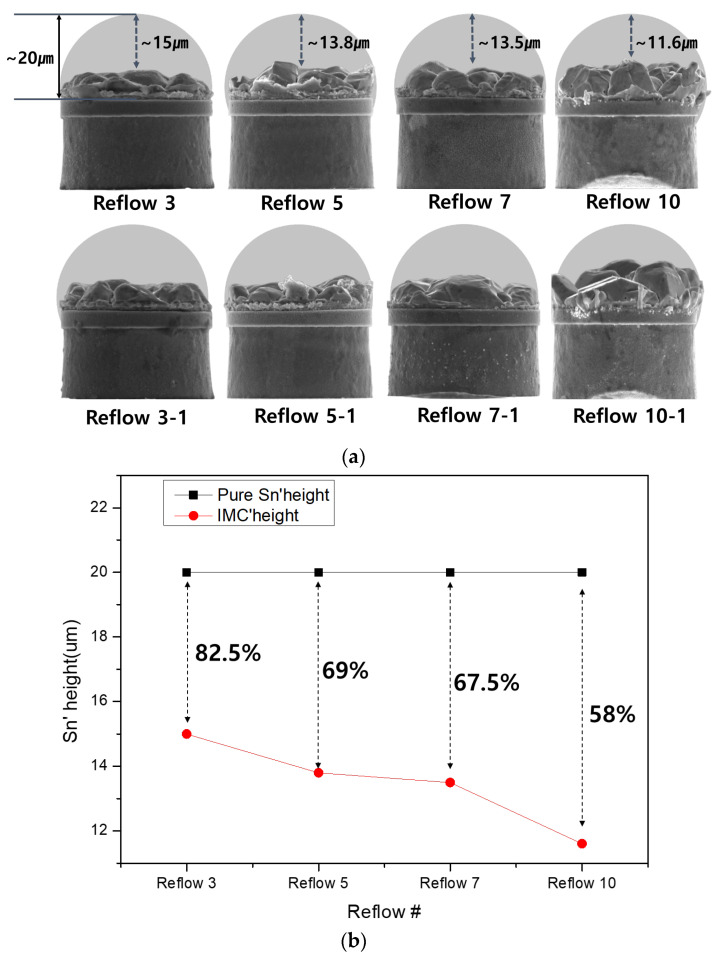
IMC analysis for the side surface of bumps according to reflow cycle. (**a**) The side surface shape of IMC according to number of reflow cycles (calculation example = (15/20) * 100, pure Sn’ height: 20 μm) and (**b**) reduction in the height of pure Sn according to the number of reflows.

Figure 12a,b quantify the growth of the thickness and roughness of IMC in the height direction of the side surface based on a height of 20 μm of pure Sn. In the case of three reflows, when the Sn bump height was 20 μm, the IMC height was analyzed to be 5.081 μm on average. The height of the remaining Sn when the height of the IMC was subtracted from the total height was calculated to be approximately 82.5%. With five reflows, the IMC height was 6.208 μm on average, and the height of Sn when the height of the IMC is subtracted was calculated to be approximately 69%. In the case of seven reflows, when the Sn bump height is 20 μm, the IMC height was 6.541 μm on average, and the height of Sn when the height of the IMC is subtracted was approximately 67.5%. In the case of 10 reflows, when the Sn bump height is 20 μm, the IMC height was 9.450 μm on average, and the height of Sn when the height of the IMC is subtracted was approximately 58%. In particular, after 10 reflows, samples were analyzed in which only about 50% or more of the Sn height remained. Furthermore, alloyed Sn and Cu growth near the Ni barrier was observed both upward and to the left and right. If the Ni barrier was absent, the growth characteristics of the IMC in the plane and the side surface would be significantly different. When interconnecting using microbumps, the remaining area of pure Sn is expected to affect electrical and long-term reliability.

## 4. Conclusions

The inter-connection method of 2.5D/3D packages such as HBM mainly applies a solder cap pure Sn for microbump. For the pure Sn microbump used in the package of the fine pitch, the IMC is necessarily formed according to the number of reflow. The IMC generated between the fine pitch bumps has a great influence on the shear strength. This effect can also be a criterion for evaluating reliability according to electrical and physical properties.

Against this background, this study focused on verifying the behavior of the size and area of IMC grains according to reflow for pure Sn microbumps formed by plating on the Ni barrier. Additionally, when the Ni barrier was formed between the Cu pillar and pure Sn, the growth behavior of IMC grain was evaluated. In order to find the IMC growth behavior of the bump, a method of removing solder cap pure Sn through etching was applied, and the behavior of IMC grain was evaluated through the results for each sample. SupraBond RO-22 had a great help in directly confirming that IMC grain was formed inside. If the Ni barrier consists of between Cu pillar and pure Sn, it will be very helpful to predict the thickness and size of IMC grain according to reflow.

First, the IMC grain roughness (Sa) formed on the bump increases almost three times linearly, from 0.370 μm in reflow 1 to 1.126 μm in reflow 10. This can be confirmed by increasing the depth between grains because of the merge of the IMC grains.

Second, the average surface area of the IMC grain formed under each condition was increased proportionally as a whole. In the case of reflow 10, the surface area was found to be about four times larger than that of reflow 1.

Third, the number of IMC grains decreased rapidly proportionally from 1 to 5, but decreased gradually from 7 to 10 reflows.

Fourth, it was evaluated how much IMC occupies for the soldier cap pure Sn with a diameter of 30 μm. In particular, it was confirmed that only about 50% or more of the pure Sn height remained after 10 reflows.

For solder cap pure Sn with a diameter of 30 μm microbumps, no results on IMC grain with or without a Ni barrier have been reported. In the future, if the behavior of IMC grain is compared for the two cases, it is judged that the importance of the Ni barrier can be confirmed through this. The etching method and analysis presented in this study are expected to be of great help in designing the electrical properties and reliability of fine pitch microbumps on the semiconductor package substrate.

## Figures and Tables

**Table 1:** Reflow process condition for each sample.

	Sn Bump Sample 1	Sn Bump Sample 2	Sn Bump Sample 3	Sn Bump Sample 4	Sn Bump Sample 5
Diameter and height of solder cap Sn	Diameter: 30 μm, Height: approx. 20 μm				
Number of reflow cycles	1	3	5	7	10

**Table 2:** Atomic composition analysis on the plane of the Sn-etched microbump.

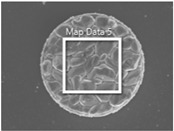	**Bump Map Sum Spectrum on Etched Sn**	
	**Element**	**wt%**
	Ni	1.10
	Cu	40.56
	Sn	58.34
	Total	100.00

**Table 3:** The intergranular roughness of the IMC grain.

	Type	#1	#2	#3	#4	#5	Average
Reflow 1 time	Sa (μm)	0.280	0.437	0.402	0.356	0.353	0.370
	Sz (μm)	2.429	4.204	3.411	3.485	3.547	3.481
Reflow 3 times	Sa (μm)	0.597	0.555	0.576	0.795	0.683	0.619
	Sz (μm)	5.273	4.425	5.063	5.776	4.909	5.081
Reflow 5 times	Sa (μm)	0.728	0.707	0.750	0.732	0.788	0.737
	Sz (μm)	6.484	4.929	6.137	6.003	6.355	6.208
Reflow 7 times	Sa (μm)	0.887	0.798	0.944	1.025	1.001	0.944
	Sz (μm)	6.917	6.012	5.835	6.646	6.872	6.541
Reflow 10 times	Sa (μm)	1.028	1.205	1.242	1.033	1.140	1.126
	Sz (μm)	7.790	9.725	8.136	8.645	9.981	9.450

**Table 4:** Number of IMC grains.

(Unit: ea)	Sample 1	Sample 2	Sample 3	Average
1 Reflow	34	39	37	36.67
3 Reflow	24	21	23	22.67
5 Reflow	13	14	15	14
7 Reflow	10	10	10	10
10 Reflow	6	6	7	6.33

**Table 5:** The surface area analysis of grains.

(Unit: μm^2^)	Sample 1	Sample 2	Sample 3	Average
1 Reflow	18.493	18.865	17.372	18.243
3 Reflow	33.012	24.428	27.565	28.335
5 Reflow	57.626	40.221	45.280	47.669
7 Reflow	61.286	57.313	59.918	59.506
10 Reflow	93.033	60.630	75.631	76.431

## Data Availability

No new data were created or analyzed in this study.

## Abbreviations

IMC	Intermetallic compound
BGA	Ball grid arrays
CSP	Chip scale packages
HBM	Bandwidth memory
RAM	Random access memory
DRAM	Dynamic random access memory
CSA	CuSnAg
ENEPIG	Electroless nickel electroless palladium immersion gold
